# On Synchronizing Coupled Retinogeniculocortical Pathways: A Toy Model

**DOI:** 10.1155/2018/6858176

**Published:** 2018-03-08

**Authors:** B. L. Mayer, L. H. A. Monteiro

**Affiliations:** ^1^Escola de Engenharia, Universidade Presbiteriana Mackenzie, Rua da Consolação, No. 896, 01302-907 São Paulo, SP, Brazil; ^2^Departamento de Engenharia de Telecomunicações e Controle, Universidade de São Paulo, Av. Prof. Luciano Gualberto, Travessa 3, No. 380, 05508-900 São Paulo, SP, Brazil

## Abstract

A Newman-Watts graph is formed by including random links in a regular lattice. Here, the emergence of synchronization in coupled Newman-Watts graphs is studied. The whole neural network is considered as a toy model of mammalian visual pathways. It is composed by four coupled graphs, in which a coupled pair represents the lateral geniculate nucleus and the visual cortex of a cerebral hemisphere. The hemispheres communicate with each other through a coupling between the graphs representing the visual cortices. This coupling makes the role of the corpus callosum. The state transition of neurons, supposed to be the nodes of the graphs, occurs in discrete time and it follows a set of deterministic rules. From periodic stimuli coming from the retina, the neuronal activity of the whole network is numerically computed. The goal is to find out how the values of the parameters related to the network topology affect the synchronization among the four graphs.

## 1. Introduction

Unveiling how nervous systems perform cognitive and sensory functions depends on understanding how stimuli from the outside world are translated into neuronal responses. For instance, in mammalian visual system, synchronized responses underlie image perception [1–8]. In fact, in mammals, visual stimulations evoke synchronous neuronal activities in retina, lateral geniculate nucleus (LGN) of the thalamus, and visual cortex (VC), at a time scale of tens of milliseconds [1–8]. For static stimuli, typical synchronization frequencies detected in these three structures are 60–120 Hz; for dynamic stimuli, synchronized oscillations in retina and LGN also occur at 60–120 Hz, but in VC at 30–60 Hz [3, 7, 8]. Therefore, the cortical frequency band is usually equal to or lower than the retinal/thalamic frequency band.

Frequency transitions in a single retinogeniculocortical pathway were already numerically investigated [9]. Here, we consider that the visual system of mammals is indeed composed by two pathways coupled by the corpus callosum, which connects the cerebral hemispheres. The aim is to examine how the amount of links connecting these neuronal structures influences the emergence of synchronized responses in LGN and VC, with equal or distinct frequencies.

As the brain has a modular architecture [10], there are many theoretical studies on neuroscience based on coupled neural oscillators [11–14]. In this work, Newman-Watts random graphs [15] are used to represent the network topology of the oscillators composing the visual pathways; and the discrete time evolution of the states of these oscillators is governed by deterministic rules, as in other models on neurodynamics [16–19].

This manuscript about synchronization of oscillatory neuronal responses is organized as follows. In Section 2, the model is described. In Section 3, results obtained from numerical simulations are presented and discussed. In Section 4, the conclusions are stressed.

## 2. The Model

The whole network of our toy model is created as follows. First, consider a square lattice with *N* rows and *N* columns, in which the *N*^2^ = *n* nodes are linked in a cross-like coupling pattern. Thus, each node has four regular neighbors (except the nodes placed at the boundaries, which have only two or three neighbors) and there are 2*N*(*N* − 1) regular edges. Then, *m* extra edges are randomly included. Obviously, the higher the value of *m*, the lower the average shortest path length *ℓ*. For instance, for *N* = 10, then *ℓ* = 6.67 for *m* = 0, *ℓ* = 5.81 ± 0.17 for *m* = 3, *ℓ* = 4.95 ± 0.13 for *m* = 9, *ℓ* = 3.99 ± 0.10 for *m* = 27, and *ℓ* = 3.14 ± 0.03 for *m* = 81. Since this Newman-Watts-type graph has small-world features [15, 20], it can be suitable to model biological neural structures [21, 22]. Hence, this undirected graph with *N*^2^ nodes, 2*N*(*N* − 1) regular edges and *m* random edges, is used to represent the LGN. It is also used to represent the VC.

In a hemisphere, the LGN is coupled to the VC by *m*′ random edges directed from the LGN to the VC. The dynamics of this single visual pathway were already examined [9]. In this work, the hemispheres are coupled by *m*′′ undirected random edges connecting the cortices. Thus, there are two retinogeniculocortical pathways coupled by callosal connections.

**Figure 1:**
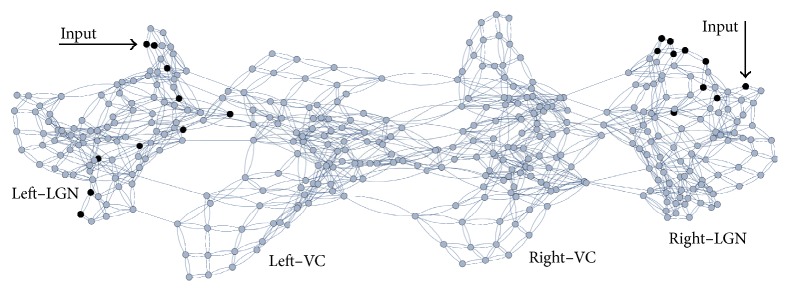
Neural network built by taking *N* = 10 and *m* = *m*′ = *m*′′ = 9. The input is applied to neurons in the first row of the lateral geniculate nuclei. These neurons are denoted by black circles. An undirected edge is represented by double line; a directed edge, by single line.

Static and dynamic visual stimuli are encoded as coherent oscillations by the ganglion retinal cells, which are the output neurons of retina [10]. Hence, periodic stimuli with period *P* coming from retinal afferents are applied to either one or all *N* nodes in the first row of the graphs representing the nuclei, as illustrated by Figure 1. Then, the activity of the whole network is determined at each time step.

At the time step *t*, each node can be in one of four states: susceptible (*S*), active-1 (*Y*), active-2 (*Z*), or inhibited (*R*). The input of *i*th node at *t* is given by ∑_*j*_*w*_*ij*_*o*_*j*_(*t*) + *I*_*i*_(*t*), in which the index *j* labels the nodes linked to the *i*th node, *w*_*ij*_ is the synaptic weight connecting the *j*th to the *i*th node, *o*_*j*_(*t*) is the output of the *j*th node, and *I*_*i*_(*t*) denotes the stimulus coming from the retina (which can be nonnull only for nodes in the first row of the nuclei). If the *j*th node is active (i.e., if it is in the state *Y* or *Z*) at *t*, then *o*_*j*_(*t*) = 1; otherwise, *o*_*j*_(*t*) = 0.

The time evolution of the activity of this neural network is driven by the following rules of state transitions [9]. A *S*-node at *t* becomes a *Y*-node at *t* + 1 if the input received at *t* is equal to or greater than the threshold *T*. A *Y*-node at *t* becomes a *Z*-node at *t* + 1 if its input at *t* remains equal to or greater than *T*; otherwise, it returns to being a *S*-node. A *Z*-node at *t* becomes a *R*-node at *t* + 1 independently of its input. A *R*-node at *t* becomes a *S*-node at *t* + 1 and it is ready to fire at *t* + 2, if the input received at *t* + 1 is strong enough. Note that these rules are deterministic.

Let *x*_1_(*t*), *x*_2_(*t*), *x*_3_(*t*), and *x*_4_(*t*) be the percentages of active nodes in the left-LGN, left-VC, right-LGN, and right-VC, respectively. Let *p*_*i*_  (*i* = 1,2, 3,4) be the period of the oscillation found in *x*_*i*_(*t*) in permanent regime (i.e., after the transient phase), in a given time window (here, a time interval of 100 time steps). The whole network is considered to be synchronized if *p*_1_/*r*_1_ = *p*_2_/*r*_2_ = *p*_3_/*r*_3_ = *p*_4_/*r*_4_, in which *r*_*i*_ are integer numbers.

We assume that *w*_*ij*_ = 1; therefore, all synapses have the same weight and they are excitatory. Inhibition, however, is a feature that can be necessary for biological neural systems achieving synchronous behavior [23–25]. In this model, inhibitory effects are taken into account in the transition *Z* → *R*. Due to this transition, a node firing at *t* and *t* + 1 is inhibited at *t* + 2; that is, it is forced to inactivity at *t* + 2. The influence on the dynamics of the number *d* of consecutive time steps that a node can remain active was already explored [9]. It was found that the lower the value of *d*, the stronger the inhibitory effects; consequently, the number of simulations without synchronization increases with *d*. Here, *d* = 2.

The states of all nodes are simultaneously updated throughout a simulation. The results obtained in 1.28 × 10^6^ simulations are reported in the next section.

## 3. Simulation Results and Discussion

Consider that one time step of the simulation is equivalent to one millisecond of real time, because this is the time interval related to the state transitions *S* → *Y* and *R* → *S* in actual neurons [10]. Consider also that a stimulus with unit magnitude and period *P* = 10 ms is applied in the first row of the graphs representing the nuclei either to a single node or to all *N* nodes. The first kind of stimulus is denoted as *k* = 1; the second one, as *k* = *N*. Observe that a forcing frequency of (1/10) ms^−1^ = 100 Hz is similar to those typically recorded in retina [3, 7, 8]. Computer simulations were performed by taking *T* = 1, *N* = 10 (thus, each one of the four graphs is composed by 100 nodes) and {*m*, *m*′, *m*′′} = {3,9, 27,81}. With these amounts of random links, the percentages of thalamocortical synapses and of callosal synapses related to cortical neurons are comparable to those found in actual nervous systems, as mentioned below.

For *k* = 1 (i.e., stimulus applied to a single node in each pathway), for each triplet (*m*, *m*′, *m*′′), 100 random networks were created and all 100 combinations of possible inputs were simulated (100 combinations, because there are 10 nodes in the left hemisphere and 10 nodes in the right hemisphere that can receive the stimulus). Therefore, 6.4 × 10^5^ simulations were accomplished. For *k* = 10 (i.e., stimulus applied to 10 neurons in each pathway), for each triplet (*m*, *m*′, *m*′′), 10000 random networks were created, in order to execute the same number of simulations as for *k* = 1. Thus, another lot of 6.4 × 10^5^ simulations was run.

**Figure 2:**
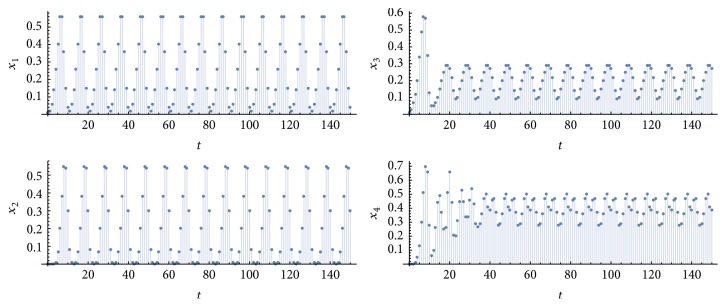
Time evolutions of *x*_1_(*t*), *x*_2_(*t*), *x*_3_(*t*), and *x*_4_(*t*), which are the percentages of active nodes in the left-LGN, left-VC, right-LGN, and right-VC, respectively. Parameter values: *T* = 1, *N* = 10, *k* = 1, *P* = 10, *m* = *m*′ = 9, and *m*′′ = 27. Observe that, in the four random graphs, the percentages tend to a periodic oscillation with period equal to *P*.

**Figure 3:**
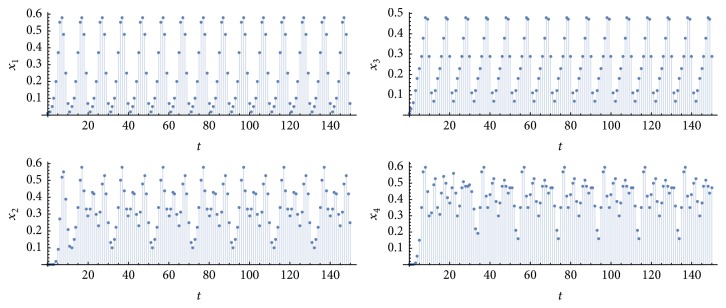
Time evolutions of *x*_1_(*t*), *x*_2_(*t*), *x*_3_(*t*), and *x*_4_(*t*). Parameter values: *T* = 1, *N* = 10, *k* = 1, *P* = 10, *m* = 9, and *m*′ = *m*′′ = 27. In this case, *p*_1_ = *p*_3_ = 10 and *p*_2_ = *p*_4_ = 20.

**Figure 4:**
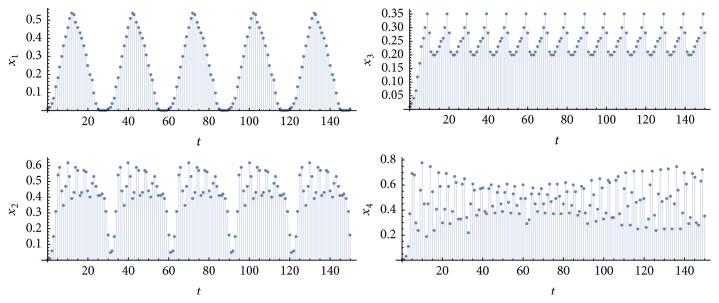
Time evolutions of *x*_1_(*t*), *x*_2_(*t*), *x*_3_(*t*), and *x*_4_(*t*). Parameter values: *T* = 1, *N* = 10, *k* = 1, *P* = 10, *m* = 3, and *m*′ = *m*′′ = 81. In this case, *p*_1_ = *p*_2_ = 30, *p*_3_ = 10, but *x*_4_(*t*) did not achieve a periodic behavior in the considered time window. Thus, the whole network did not synchronize.

**Figure 5:**
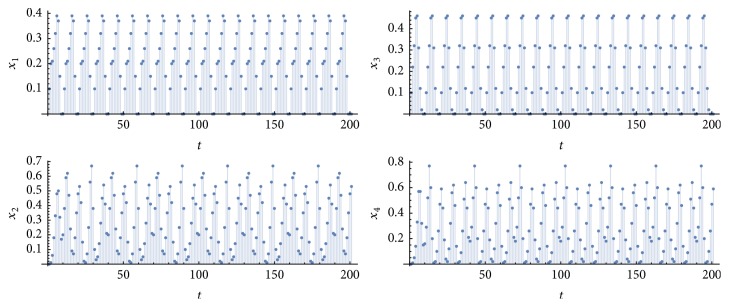
Time evolutions of *x*_1_(*t*), *x*_2_(*t*), *x*_3_(t), and *x*_4_(*t*). Parameter values: *T* = 1, *N* = 10, *k* = 10, *P* = 10, *m* = *m*′ = 9, and *m*′′ = 27. In this case, *p*_1_ = *p*_3_ = 10 and *p*_2_ = *p*_4_ = 30.

Let *η*_1_^*k*^ be the number of simulations in which *P* = *p*_1_ = *p*_2_ = *p*_3_ = *p*_4_ = 10 and *η*_2_^*k*^ is the number of simulations with *P* = *p*_1_ = *p*_3_ = 10 and *p*_2_ = *p*_4_ = 20 or 30, for *k* = {1,10}.  Figure 2 shows an example of a simulation of type-*η*_1_^1^ (retina, nuclei, and cortices oscillating at the same frequency 1/*P*, due to a periodic input of period *P* applied to a single neuron in each pathway), Figure 3, an example of type-*η*_2_^1^ (retina and nuclei at the frequency 1/*P*, and cortices at a frequency that is half or a third of 1/*P*).  Figure 4 exhibits an example in which synchronization did not occur; that is, at least one graph did not achieve a periodic behavior in the time window of 100 time steps (100 milliseconds of real time).  Figure 5 illustrates the case *η*_2_^10^.

In order to evaluate the influence of the topological parameters *m*, *m*′, and *m*′′, we proceeded as follows. For each value of *m*, we added the numbers corresponding to *η*_1_^*k*^ and *η*_2_^*k*^ obtained for all combinations of *m*′ and *m*′′. For instance, for *m* = 3  e  *k* = 1, we added the numbers of *η*_1_^1^ obtained in 160000 simulations with *m*′ = {3,9, 27,81} and *m*′′ = {3,9, 27,81}; then, we added the numbers corresponding to *η*_2_^1^. A similar procedure was carried out for evaluating the influences of *m*′ and *m*′′ on the synchronization of the whole network.

Table 1 presents the results. The first obvious observation is that *η*_1_^10^ > *η*_1_^1^ for any parameter values. Thus, input applied to *k* = 10 neurons in each pathway enhances the number of cases in which the whole network is synchronized at the frequency 1/*P*, as compared to input applied to only *k* = 1 neuron. The relation *η*_2_^10^ > *η*_2_^1^ is also valid for most cases (it does not hold only for *m* = 81). Thus, the higher the number of nodes in LGN receiving the input, the higher the number of synchronized networks.

About the influence of *m*′′, which is the number of callosal links connecting the cortices: *η*_1_^*k*^ tends to decrease and *η*_2_^*k*^ tends to increase with *m*′′. This effect is more pronounced for *k* = 10 than for *k* = 1. Therefore, VC synchronously oscillating at a lower frequency than LGN can be favored by increasing both *m*′′ and the number of nodes in LGN receiving the input.

About the influence of *m* and *m*′, which are, respectively, the amount of random links inside each graph and the amount of thalamocortical links: the dependencies of *η*_1_^*k*^ and *η*_2_^*k*^ on *m* and *m*′ are not monotonous. Note that the maximum numbers of *η*_1_^*k*^ are found for extreme values of *m* and *m*′: for *k* = 1 and *k* = 10, the maxima occur for *m* = 81 and *m*′ = 3. The maximum numbers of *η*_2_^*k*^ occur for intermediate values of *m* and *m*′: for *k* = 1, the maxima are found in *m* = 27 and *m*′ = 27; for *k* = 10, in *m* = 27 and *m*′ = 9.

Take *N* = 10 and *m* = *m*′ = *m*′′ = 27. Thus, there are 4*N*(*N* − 1) + 2*m* + 2*m*′ + *m*′′ = 495 connections involving cortical nodes. In this network, the percentage of thalamocortical synapses is 54/495≃11%, and the percentage of callosal synapses is 27/495≃5%. Interestingly, similar values can be found in literature. For instance, in mouse cortex, thalamocortical synapses are about 15% [26]; callosal synapses, about 5% [27]. One can conjecture that the values of *m*, *m*′, and *m*′′ found in actual visual pathways favor the occurrence of synchronization between LGN and VC, in order that the frequency observed in VC is equal, half, or a third of the one measured in LGN.

In our toy model, for obtaining synchronization between LGN and VC in distinct frequencies, the random links connecting them must be directed. If undirected links are used to connect LGN and VC, simulations show that either the four graphs synchronize with period equal to *P* or they do not synchronize.

## 4. Conclusions

Toy models can be valuable, because they can reveal which features of the studied system are most relevant. Here, a toy model was employed to simplistically investigate the dynamics of coupled visual pathways. Only three neural structures were taken into consideration: retina (represented as a periodic input), LGN, and VC (both represented by Newman-Watts graphs). The aim was to determine how the wiring topologies affect their synchronous activities, because synchronization among these neural structures is supposed to be underlying the visual perception [1–8].

Callosal connections can be responsible for integrating interhemispheric information, binding perceptual features and enabling a coherent representation of a visual scene [28]. We found that *η*_2_^*k*^ significantly increases with *m*′′ when the stimulus is applied to all nodes in the first row of both pathways; if it is a applied to a single node, then *m*′′ barely affects the synchronization. Thus, interhemispheric synchronization can be improved not only by increasing *m*′′, but also by increasing the number of stimulated nodes. It is interesting to note that *η*_1_^*k*^ tends to decrease with *m*′′. Thus, increasing the amount of callosal connections favors that the cortices synchronously oscillate in a frequency lower than the one detected in the nuclei.

A key component of the neural coding is the timing of spiking activity [29]. This timing is influenced by the neuron features and by the synaptic connectivity [30, 31]. In this context, our model shows that there are values of *m* and *m*′ favoring the cases labeled as *η*_1_^*k*^ and *η*_2_^*k*^. For instance, according to Table 1, the maxima of *η*_2_^1^ and *η*_2_^10^ occur for *m* = 27. By taking *m* = *m*′ = *m*′′ = 27, then 11% of synapses involving cortical neurons are thalamocortical ones and 5% are callosal ones, which are values compatible with those found in actual nervous systems [26, 27]. Notice that the validity of our results relating the amounts of synaptic connections (the parameters *m*, *m*′, and *m*′′) to synchronous behaviors classified as *η*_1_^*k*^ and *η*_2_^*k*^ can be experimentally tested.

## Figures and Tables

**Table 1:** Number of cases classified as *η*_1_^1^, *η*_2_^1^, *η*_1_^10^, and *η*_2_^10^ in function of *m*, *m*′, and *m*′′ for coupled visual pathways with *T* = 1, *N* = 10, and *P* = 10. In each line, *η*_1_^*k*^ + *η*_2_^*k*^ < 160000, because no synchronization and synchronization with period relationships distinct from those labeled as *η*_1_^*k*^ and *η*_2_^*k*^ were found.

	*η*_1_^1^	*η*_2_^1^	*η*_1_^10^	*η*_2_^10^
*m* =				
3	1206	311	130588	16947
9	21308	5211	136631	19991
27	112597	24083	133960	25606
81	139869	19728	141031	18905

*m*′ =				
3	78265	3417	151341	4898
9	62755	18681	112136	39408
27	58819	22241	129501	28236
81	75141	4994	149232	8907

*m*′′ =				
3	69249	11295	138688	17440
9	69843	12299	136990	18552
27	68512	12836	134758	21170
81	67376	12903	131774	24287
